# Transcriptional Responses of *Dictyostelium discoideum* Exposed to Different Classes of Bacteria

**DOI:** 10.3389/fmicb.2020.00410

**Published:** 2020-03-10

**Authors:** Otmane Lamrabet, Astrid Melotti, Frédéric Burdet, Nabil Hanna, Jackie Perrin, Jahn Nitschke, Marco Pagni, Hubert Hilbi, Thierry Soldati, Pierre Cosson

**Affiliations:** ^1^Department of Cell Physiology and Metabolism, Faculty of Medicine, University of Geneva, Geneva, Switzerland; ^2^Vital-IT Group, SIB, Swiss Institute of Bioinformatics, Lausanne, Switzerland; ^3^Department of Biochemistry, Faculty of Science, University of Geneva, Geneva, Switzerland; ^4^Faculty of Medicine, Institute of Medical Microbiology, University of Zurich, Zurich, Switzerland

**Keywords:** *Dictyostelium discoideum*, host-pathogen interactions, RNA-seq, *Klebsiella pneumoniae*, *Bacillus subtilis*, *Mycobacterium marinum*, folate, *Micrococcus luteus*

## Abstract

*Dictyostelium discoideum* amoebae feed by ingesting bacteria, then killing them in phagosomes. Ingestion and killing of different bacteria have been shown to rely on largely different molecular mechanisms. One would thus expect that *D. discoideum* adapts its ingestion and killing machinery when encountering different bacteria. In this study, we investigated by RNA sequencing if and how *D. discoideum* amoebae respond to the presence of different bacteria by modifying their gene expression patterns. Each bacterial species analyzed induced a specific modification of the transcriptome. Bacteria such as *Bacillus subtilis, Klebsiella pneumoniae*, or *Mycobacterium marinum* induced a specific and different transcriptional response, while *Micrococcus luteus* did not trigger a significant gene regulation. Although folate has been proposed to be one of the key molecules secreted by bacteria and recognized by hunting amoebae, it elicited a very specific and restricted transcriptional signature, distinct from that triggered by any bacteria analyzed here. Our results indicate that *D. discoideum* amoebae respond in a highly specific, almost non-overlapping manner to different species of bacteria. We additionally identify specific sets of genes that can be used as reporters of the response of *D. discoideum* to different bacteria.

## Introduction

*Dictyostelium discoideum* is a free-living amoeba, and a well-established model organism for the study of basic aspects of differentiation, signal transduction, phagocytosis, cytokinesis and cell motility (Cosson and Lima, 2014; Nichols et al., 2015; Dunn et al., 2017). In its natural habitat, the forest soil, this professional phagocyte feeds upon bacteria that are then killed in phagosomes. Under controlled conditions, *D. discoideum* can feed upon a large number of bacterial species (Raper, 1935, 1936). Yet to the best of our knowledge, the amoeba uses different molecular mechanisms to kill different species of bacteria: For example, the Kil1 and Kil2 proteins ensure efficient intracellular killing of *Klebsiella pneumoniae* but are not involved in killing of *Bacillus subtilis* (Benghezal et al., 2006; Lelong et al., 2011). It would thus seem consistent that *D. discoideum* responds specifically to the presence of different bacteria by adapting its gene expression pattern. On the other hand, *D. discoideum* is also the target of pathogenic bacteria (Reviewed in Dunn et al., 2017 and Cardenal-Muñoz et al., 2018) and has been used as a model host to identify and study bacterial virulence factors (Cosson et al., 2002; Bozzaro and Eichinger, 2011; Koliwer-Brandl et al., 2019). It has also been suggested that amoebae serve as environmental reservoir for certain human pathogens (Greub and Raoult, 2004). Accordingly, one would expect *D. discoideum* to adapt to the presence of pathogenic bacteria in order to survive their encounter. Finally, some bacterial pathogens manipulate the physiology of *D. discoideum* to use it as a niche for intracellular replication (Hoffmann et al., 2014; Swart et al., 2018). Little is known about how these various responses collectively modify the gene expression profile of *D. discoideum* when it encounters various bacteria. Similarly, the molecular mechanisms linking exposure to bacteria with alterations in gene expression are still mostly unknown.

At the molecular level, the *D. discoideum* genome encodes 61 putative G-protein-coupled receptors (GPCRs), including 17 γ–aminobutyric acid (GABA) or metabotropic glutamate receptor-like proteins, known as Grl (glutamate receptor-like) proteins (Heidel et al., 2011). GrlL and GrlG have recently been proposed to act as receptors allowing *D. discoideum* cells to respond to folic acid, which is released from bacteria (Pan et al., 2018; Thomas et al., 2018). *D. discoideum* amoebae do not have Toll-like receptors orthologs (Cosson and Soldati, 2008; Dunn et al., 2017). However, two cytosolic proteins with Toll/Interleukin1-Receptor (TIR) domains potentially involved in intracellular signaling, TirA and TirB, have been identified (Chen et al., 2007). The *D. discoideum* genome encodes more than 100 proteins containing leucin-rich repeats (LRR), but whether they function as pattern recognition receptors remains to be determined (Basu et al., 2013). Overall, the *D. discoideum* genome exhibits numerous genes that could allow it to recognize specifically various types of bacteria, but the specific role of individual gene products remain to be defined.

In recent years, several transcriptomic studies have revealed that specific metabolic and signaling pathways are upregulated when *D. discoideum* cells feed on different bacteria (Farbrother et al., 2006; Carilla-Latorre et al., 2008; Sillo et al., 2008; Nasser et al., 2013). This specific modulation probably reflects at least in part the fact that each bacterial species contains different nutrients. In addition, it is expected that *D. discoideum* recognizes on bacteria specific, conserved features (microbe-associated molecular patterns, MAMPs), and as a consequence, activates specific intracellular signaling pathways. Accordingly, transcriptional responses of amoebae exposed to bacteria most likely reflect a combination of metabolic adaptation and specific recognition. In each of the experiments reported the authors used different experimental setups that would induce metabolic adaptation and specific recognition to different extents. For example, in some studies *D. discoideum* amoebae were fed exclusively with non-pathogenic bacteria for several generations (16 h) (Nasser et al., 2013) or for a shorter time (2 h) (Sillo et al., 2008). In other studies, *D. discoideum* amoebae were exposed to pathogenic *Legionella pneumophila* (Farbrother et al., 2006), *Pseudomonas aeruginosa* (Carilla-Latorre et al., 2008), or *Mycobacterium marinum* (Hanna et al., 2019). These experimental variations, as well as the use of widely different techniques presumably account for the fact that very diverse results were obtained.

In this study, we analyzed the transcriptional response of *D. discoideum* cells exposed to different bacteria, or to folate, in rich medium, to minimize metabolic adaptation. Our results indicate that even in this experimental setup, *D. discoideum* amoebae respond in a highly specific manner to different species of bacteria both quantitatively and qualitatively. This study also identified sets of genes that can be used to test whether specific *D. discoideum* mutants respond to various stimuli.

## Materials and Methods

### Cell Culture and Strains

*Dictyostelium discoideum* DH1 cells were cultured in HL5c medium (Formedium) at 21°C and subcultured twice a week to maintain a cellular density below 10^6^ cells/mL.

Bacterial strains were grown overnight in LB medium at 37°C. Bacteria used were the non-pathogenic *K. pneumoniae* KpGe laboratory strain (KpGe) (Lima et al., 2018), fluorescent *K. pneumoniae* KpGe expressing yEGFP (Bodinier et al., 2020), the pathogenic LM21 strain of *K. pneumoniae* (Kp21) (Favre-Bonte et al., 1999), non-sporulating *B. subtilis* 36.1 (Bs) (Ratner and Newell, 1978), a flagella-less *B. subtilis* expressing mCherry (Bodinier et al., 2020), *Micrococcus luteus* (Ml) (Wilczynska and Fisher, 1994), and pathogenic *M. marinum* M strain (Mm) (Volkman et al., 2004).

### RNA Extraction for RNA-Seq

Wild-type *D. discoideum* cells (4 × 10^6^) were washed and resuspended in 1 mL of HL5c medium containing 15 μg/mL of tetracycline to prevent bacterial growth. Bacteria were added at a multiplicity of infection (MOI) of 500. Alternatively, 1 mM of folate was added. The cells were then incubated at 21°C at 120 rpm for 4 h. As a control, *D. discoideum* cells were cultured separately in HL5c medium containing 15 μg/mL of tetracycline.

After co-incubation, the uningested bacteria were removed by centrifugation at 1000 *g* for 2 min, RNA was extracted from cells using the direct-zol RNA extraction kit (Zymo research) following the manufacturer’s instructions for total RNA isolation. To remove contaminating genomic DNA, samples were treated with 0.25U of DNase I (Zymo) per 1 μg of RNA for 15 min at 25°C. RNA was quantified using Qubit 4.0 (Invitrogen) and its quality was checked using an Agilent 2100 Bioanalyzer (Agilent Technologies).

### Construction of cDNA Libraries and RNA Sequencing

The cDNA preparation and the RNA sequencing were performed as described previously (Hanna et al., 2019). Briefly, the total RNA preparation was used as a template for cDNA synthesis and NGS library construction using the Ovation Universal System (NuGEN Technologies, San Carlos, CA, United States). 100 ng of total DNAse I-treated RNA was used for first- and second-strand cDNA synthesis following the manufacturer’s protocol. In order to obtain a comparable library size, a double bead cut strategy was applied using the 10X genomics protocol. cDNA was recovered using magnetic beads with two ethanol washing steps, followed by enzymatic end repair of the fragments. Barcoded adapters were ligated to each sample before strand selection. Ribosomal RNAs were targeted for depletion by the addition of custom-designed oligonucleotides specific for *D. discoideum* (5S, 18S, and 28S). To amplify the libraries, 18 cycles of PCR were performed.

The quality of the libraries was monitored by TapeStation (Agilent, High Sensitivity D1000 ScreenTape, # 5067–5584). Samples were pooled in approximately equimolar amounts and analyzed in 50 bp single read flow cells (Illumina, # 15022187; Hiseq 4000).

### Reverse-Transcription Quantitative PCR (qRT-PCR)

*Dictyostelium discoideum* cells were incubated in HL5c medium as described above in the presence or absence of bacteria. *D. discoideum* RNA was purified with a Qiagen RNeasy kit following the manufacturer’s instructions. cDNA was synthesized from 1 μg of total RNA using random hexamers and Superscript II reverse transcriptase (Invitrogen). Oligonucleotides specific for each gene tested were designed by Primer3 software (Untergasser et al., 2012). Sequences were aligned against the *D. discoideum* coding sequence database by BLAST to ensure that they were specific for the gene tested. Table 1 shows a list of all genes analyzed.

**TABLE 1:** Genes and primers used for qRT-PCR transcriptional profile analysis.

**Genes**	**DDB_G number**	**Gene name**	**Forward primer**	**Reverse primer**
Control1	DDB_G0294034	*rnlA*	GCACCTCGATGTCGGCTTAA	CACCCCAACCCTTGGAAACT
Control2	DDB_G0275153	*gpdA*	GGTTGTCCCAATTGGTATTAATGG	CCGTGGGTTGAATCATATTTGAAC
KB1	DDB_G0273235	–	CACAACATGTTGTTCTTCACC	GTCCATCACTTTCAACACCAC
KB2	DDB_G0277839	*cxgS*	CGTTCAAGATATTGGAGTTGC	CATAGAAAGCAACTCTTTCTC
KB3	DDB_G0285345	–	TTTGGCTGTCGTATTGACCGG	GCCCATAGTGGTATTTCTGTC
KB4	DDB_G0272244	*grlG*	CGTTGACGGCTATAATGATACC	CTTTACAAGCTTCTACATGTCC
K1	DDB_G0270060	*–*	TTAGAAAGCCAGTTGAAAGAG	CCATATCCTCCACTCTTATC
K2	DDB_G0275689	*abcG2*	GCAGCTGATAATACAATTGGC	TCTAGCGGTGACATACATTCC
K3	DDB_G0289575	*–*	GTGGTGTTGTAAATTCTTTAGG	TGGCAGTACCACATGTTCCAT
K4	DDB_G0279493	*–*	TGATGAAGACCCCCTTGAAAC	CTGAGTATGAATATCCATTTGC
K5	DDB_G0273217	*–*	GACAATTGTTCATTGCAACCC	CACTGTTCCATCTTCGTTAC
K6	DDB_G0273389	*psiG*	GTTCAGAAGTAACAAAAAGTCG	GTTGGACTATCACCTTCCAA
K7	DDB_G0286717	*ponC1*	CTTCTTCAAACGCAGCTGAAAC	GCAGATTGGCAAGTATTAACC
K8	DDB_G0270022	–	ATCTCAACCTCAAAGTGAAGC	GTAGTTGTTGTAGTTGTTGG
B1	DDB_G0294589	–	CACAGGAACATCAAGTGGCA	CTGGTCTACGAGTTAATGCTG
B2	DDB_G0267774	*abkC*	GGGTTTAATATTGTTACCGAG	TTATCCAACATGTACCACTG
B3	DDB_G0288095	–	TTTCTCAAGAGGGAGCTTAC	CCTGATTGATCTGGTAATGAC
B4	DDB_G0280335	*–*	CCGATTCAAATTCTGGTTC	CGCTTTCATATCCTTCTTCC
B5	DDB_G0293662	*–*	CAGTTGGTATAATTGGAGCAG	TTCTACCACCGATTCTTTC
B6	DDB_G0282061	*–*	GTGGCATTCTCAGATGATAC	TATCCACCATCAACTCTTGC
B7	DDB_G0279683	*–*	GAGCAATTATTACTGGTGC	ATCCAGATTCTCTTCTACC
B8	DDB_G0285641	*gacJ*	GTATCACCAGCAATTCAACC	GACCTCTAGAATATTCCAC
B9	DDB_G0285391	*xacC*	CACCAACTGGTACAAGTCC	GGTGGTGGTAATTGACCAC
B10	DDB_G0291007	*gxcK*	CTTCAGTACCATCCATTTCAGCA	TGTTGGTGTTGTTGTTGTGGTG
B11	DDB_G0278813	–	GCTTCATCTTTAGAGATGGAG	TCAGATGCAGATGTGCAACC

PCR reactions (10 μL) contained SYBR Green Master Mix (Applied Biosystems), diluted cDNA (150 ng) and 500 nM of forward and reverse primers, and were analyzed in a StepOnePlus cycler (Invitrogen) with the following parameters: 95°C/1 min, 40 cycles of 95°C/10 s, and 60°C/1 min. The cycle threshold (CT) value of a reaction is defined as the cycle number when the fluorescence of a PCR product can be detected above the background signal. Fold changes were calculated as Δ(ΔCT), where ΔCT = CT (target) – CT (control genes: *gpdA* and *rnlA*) and Δ(ΔCT) = ΔCT (stimulated: bacteria) - ΔCT (control condition: no bacteria). Data were collected from three biological replicates, with three technical replicates for each condition.

### Bioinformatic Analysis

The bioinformatic analysis was performed as described previously (Hanna et al., 2019). Briefly, RNA-seq libraries of *D. discoideum* exposed to bacteria or folate were compared to *D. discoideum* incubated in HL5c. 50 nt single-end reads were mapped to the *D. discoideum* genome (downloaded from dictybase) (Fey et al., 2008) using tophat (version 2.0.13) and bowtie2 (version 2.2.4) softwares. When applicable, the same procedure was applied to map reads to the relevant bacterial genome. As the RNA-seq data is stranded, parameter library-type was set to fr-second strand. Multi hits were not allowed, by using the option – max-multi hits 1. The other parameters were set to default. The read counts per gene were generated using HTSeq software (version 0.6.1) and the GFF annotation downloaded from dictybase (February, 2019). Options for htseq-count were -t exon – stranded = yes -m union. The counts were then imported in the R software package (version 3.2.2). The genes were filtered for minimal expression, by removing genes with an average through all samples lower than five reads. Normalization factors to scale the libraries sizes were calculated using edgeR. The read counts were then log-transformed and variance stabilized using voom. The log-transformed counts were then batch-corrected for date effect using the R package limma and the removeBatchEffect function.

A differential expression analysis used the R package limma, including the date batch effect in the design. In total six comparisons were performed (Supplementary Table S1). The genes having an adjusted *p*-value (using the Benjamini–Hochberg method) lower than 0.01 and an absolute log2 fold change ratio greater or equal than 2 were considered differentially expressed (Supplementary Table S2). The union of these genes was then taken for the following analyses.

The principal component analysis was generated using the R function prcomp, with centering and scaling of the data. The data were first corrected for date-batch effect before plotting all seven subpopulations. The first four principal components were considered and plotted versus each other.

For the tSNE analysis, the Rtsne package was used to plot the expression data. Dimensions were reduced to 2, with the parameters’ perplexity = 17 and theta = 0.

### Gene Ontology Analysis

For a Gene Ontology (GO) term analysis, topGO was used (version 2.36.0), as previously described (Hanna et al., 2019). In particular, a less stringent set of thresholds values were used to filter input of differentially expressed genes for the GO analysis: a *p*-value ≤ 0.05 and an absolute log2 fold change ratio ≥0.585 (corresponding to a fold change of at least 1.5). For each comparison, upregulated and down-regulated gene sets were fed separately to topGO. First, the weight01 algorithm was used to identify the lowest level significant terms for each comparison. Then, to compare the results between each comparison, the union of these terms was used to run the classical algorithm and subsequently the Fisher’s exact test. The Fold Enrichment (FE) for the enriched GO terms was calculated in the same manner as in DAVID (Huang da et al., 2009). To select the significant GO terms, an unadjusted *p*-value cutoff of 0.05 and a FE cutoff of 2 were used.

### Genes Expression Analysis Using Transcriptional Datasets

We analyzed in the transcriptional datasets the expression of 19 genes implicated in phagocytosis and intracellular killing, 17 *grls* genes encoding G-protein coupled receptors, 18 genes potentially implicated in intracellular signaling, 30 control genes encoding for protein with unknown function and 30 control genes selected randomly from amoeba genome. We also analyzed the whole set of detected genes as an additional control. The genes having an adjusted *p*-value lower or equal to 0.01 and an absolute log2 fold change ratio greater or equal than 1 were considered differentially expressed and were used in this analysis. The average fold change corresponds to the sum of the log2 fold change values divided by the number of studied genes in all conditions. The percentage indicates the number of differentially expressed genes divided by the number of studied genes in all conditions.

## Results

### Transcriptional Response of *D. discoideum* to Various Stimuli

In order to measure changes in gene expression when *D. discoideum* encounters different bacteria, we incubated *D. discoideum* in HL5c medium, or in HL5c medium supplemented with live bacteria or with 1 mM folate. Two species of Gram-positive bacteria were used (*B. subtilis* and *M. luteus*) as well as two strains of Gram-negative *K. pneumoniae* (pathogenic Kp21 and non-pathogenic KpGe), and one pathogenic mycobacterial species (*M. marinum)*. We kept cells in rich HL5c medium under all conditions and extracted cellular RNAs after a relatively short time (4 h) in order to minimize trophic effects caused by either nutrient depletion by bacteria or by the fact that *D. discoideum* could use ingested bacteria as food sources. Tetracycline, a bacteriostatic antibiotic was present in the HL5c medium and prevented the growth of extracellular bacteria. Accordingly, no extracellular bacteria growth was observed after 4 h of coculture with *D. discoideum* cells (Supplementary Figure S1A). In addition, and as expected, we did observe that *D. discoideum* cells ingested bacteria (Supplementary Figure S1B).

**FIGURE 1:**
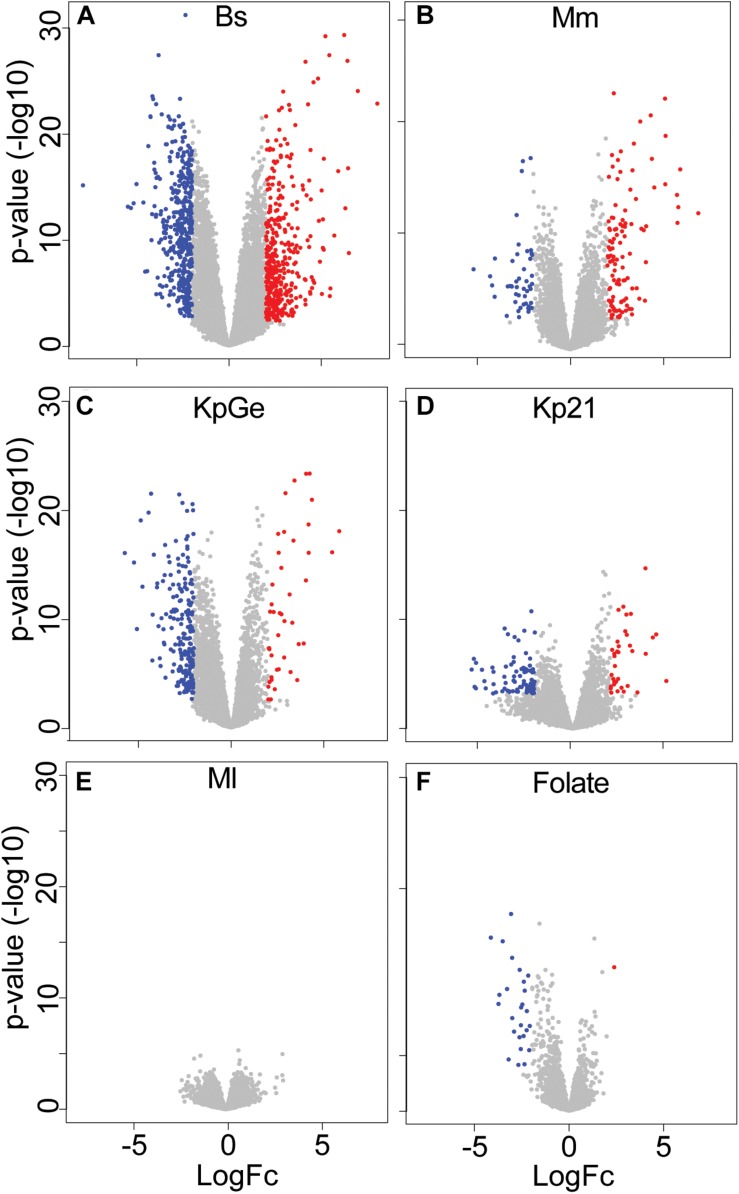
Summary of the RNA-seq analysis. Volcano plot representation of differential expression analysis of genes when the amoebae were exposed to different bacteria [**(A)** *B. subtilis* (Bs), **(B)** *M. marinum* (Mm), **(C)** *K. pneumoniae* non-pathogenic (KpGe) and **(D)** pathogenic LM21 (Kp21) strains, and **(E)** *M. luteus* (Ml)] or in the presence of folate **(F)**. Red and blue dots correspond to genes with significantly increased or decreased expression under each condition (fold changes ratio greater than 4 or less than –4 with an adjusted *p*-value ≤ 0.01). The *x*-axis shows the log2 of the fold changes of expression and the *y*-axis the adjusted *p*-value (–log10) for each gene.

We then measured the expression levels of *D. discoideum* messenger RNAs (mRNAs) by RNA sequencing (RNA-seq), using the sequence reads that mapped to unique locations in the *D. discoideum* reference genome. The expression of 8,553 genes was detected reliably under at least one condition (Supplementary Table S1). Among these, differentially expressed (DE) genes were determined by comparing expression levels in *D. discoideum* cultured in HL5c medium in the presence or absence of each bacterial species or strain. Under each condition, genes were considered to be differentially regulated if the absolute average transcript value varied at least 4-fold with an adjusted *p*-value below or equal to 0.01 (Supplementary Table S2). By this relatively strict criterium, 1,021 genes were differentially expressed under at least one condition compared to the control condition (HL5c medium). This stringent threshold was more appropriate to analyze the specificity of the response to different bacteria. Note that a less stringent selection (≥1.5-fold variation, *p*-value below or equal to 0.05) results in a significantly higher number of DE genes (see below). The number of differentially expressed genes varied markedly under different conditions, being maximal in cells exposed to *B. subtilis* (787 DE genes), high in cells exposed to *K. pneumoniae* (Kp21 and KpGe) and *M. marinum* (245, 116, and 162 DE genes, respectively), low in cells exposed to folate (27 DE genes) and null in cells exposed to *M. luteus* (Supplementary Table S2). Examination of volcano plots visualized these observations (Figure 1).

### Specificity of the Transcriptional Response of *D. discoideum* to Various Stimuli

**FIGURE 2:**
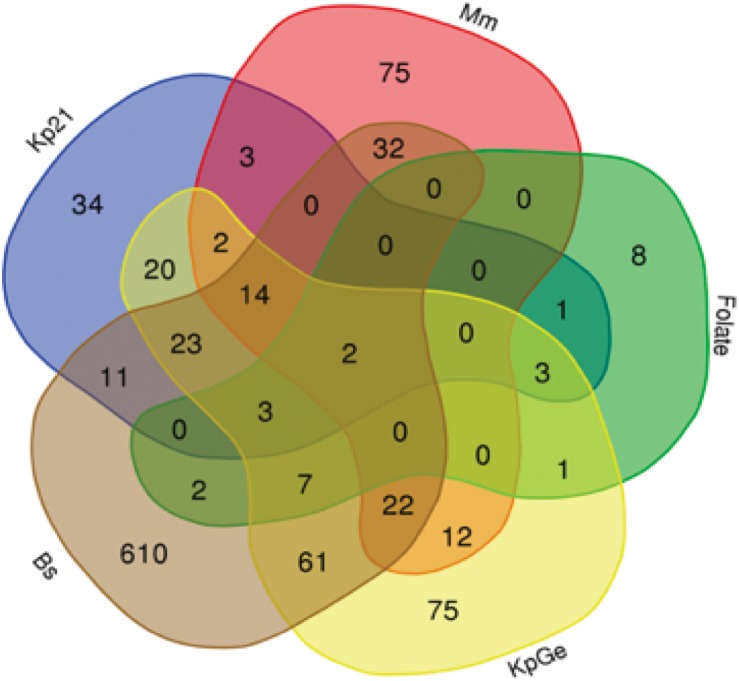
Specificity of transcriptional responses. Venn diagrams showing the number of differentially expressed genes in the presence of various bacteria: *K. pneumoniae* non-pathogenic (KpGe) and pathogenic LM21 (Kp21) strains, *B. subtilis* (Bs), *M. marinum* (Mm), and folate. The total numbers of differentially expressed genes are 787 in the presence of Bs, 245 in the presence of KpGe, 116 in the presence of Kp21, 162 in the presence of Mm, and 27 in the presence of folate (Supplementary Table S2).

To visualize the specificity of the transcriptional responses induced by different bacteria, we indicated on a Venn diagram the set of differentially regulated genes defined by the stringent criteria defined above (Figure 2). A large number of genes (802 out of 1,021) was differentially expressed only in a single condition, suggesting that the response to different stimuli was highly specific. Genes modulated only in the presence of one stimulus were numerous in the presence of *B. subtilis* (610 genes) but were also seen in cells exposed to *M. marinum* (75), *K. pneumoniae* KpGe (75), Kp21 (34), and even folate (8) (Figure 2). As a rule, genes that are differentially expressed in two different conditions vary in the same direction. For example, among the 61 genes differentially expressed in the presence of Bs and KpGe, 13 (21.5%) were upregulated and 48 (78.5%) downregulated.

### Validation of RNA-seq Results Using qRT-PCR

**FIGURE 3:**
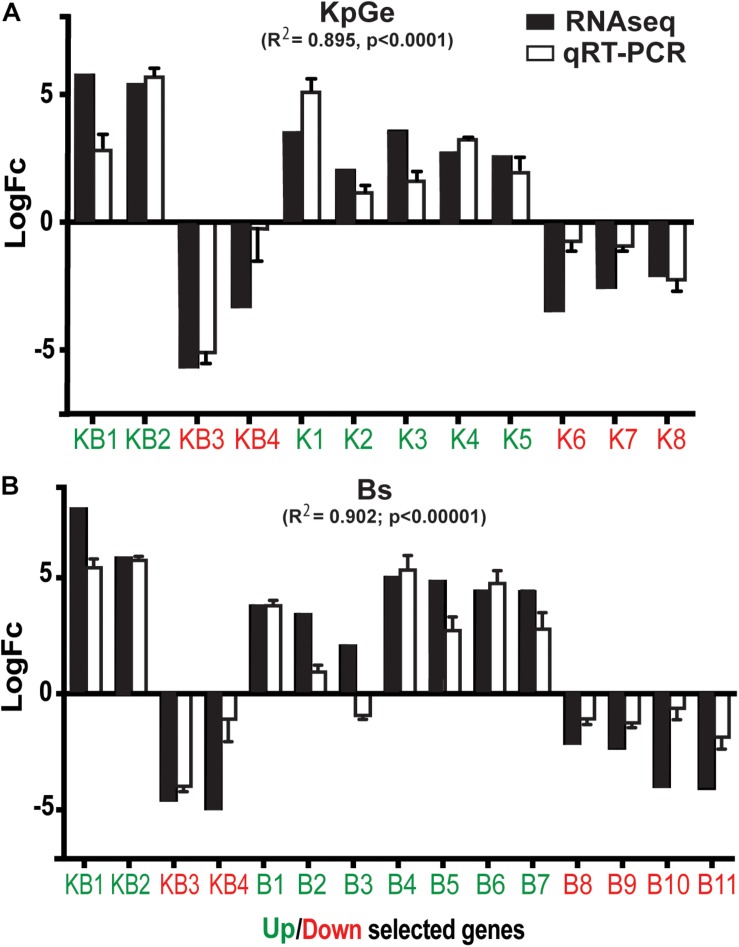
Expression and transcription profiles of selected genes. Expression profiles of selected genes observed using RNA-seq or qRT-PCR methods, when the amoebas were exposed to *K. pneumoniae* (KpGe) **(A)** or *B. subtilis* (Bs) **(B)**. 8 genes specific for the presence of KpGe (K1-8), 5 up-regulated (green) and 3 down-regulated (red); 11 genes specific for the presence of Bs (B1-11), 7 up-regulated (green), and 4 down-regulated (red); and 4 genes specific for the presence of both KpGe and Bs (KB1-4), 2 up-regulated (green) and 2 down-regulated (red). The Pearson correlation coefficients between RNA-seq and qRT-PCR results are indicated.

To validate the RNA-seq results we selected 23 genes that were up or down-regulated in cells exposed to *B. subtilis* and *K. pneumoniae* KpGe (Table 1). Of these 23 genes, 8 genes varied specifically in the presence of *K. pneumoniae* KpGe (K1-8) (5 up-regulated and 3 down-regulated), 11 in the presence of *B. subtilis* (B1-11) (7 up-regulated and 4 down-regulated), and 4 in the presence of both *K. pneumoniae* KpGe and *B. subtilis* (KB1-4) (2 up-regulated and 2 down-regulated). We then measured the expression profiles of these selected genes by reverse-transcription quantitative PCR (qRT-PCR) in three independent experiments, and compared the results with the transcriptional profiles deduced from the RNA-seq experiments (Figure 3). We observed a strong correlation between the results of RNA-seq experiments and those obtained by qRT-PCR (Pearson correlation, *R*^2^ = 0.895 and *R*^2^ = 0.902, using *K. pneumoniae* KpGe or *B. subtilis*, respectively) (Figure 3). From the 23 selected genes, 21 showed a qualitatively similar transcriptional profile using both methods and only two (KB4 and B3) did not (Figure 3).

### Global Analysis of DE Genes Reveals Transcriptional Signatures Specific for the Exposure to Each Type of Bacterium

**FIGURE 4:**
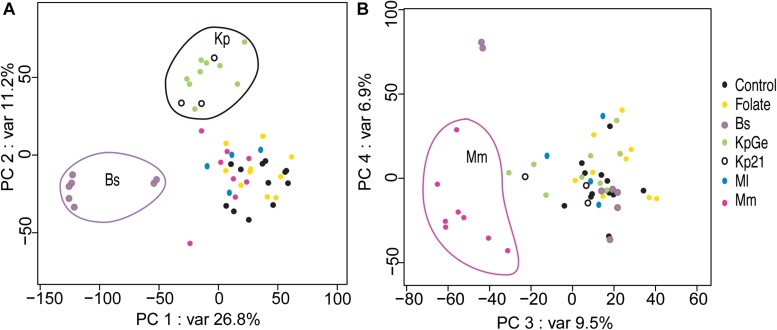
Principal component analysis. Each dot represents the expression profile of one sample in an independent experiment. The axis labels indicate the percentages of variance explained by each principal component. All analyses were performed including the experimental batch as a covariate in the statistical model. **(A)** The axes correspond to the first (*x* axis) and the second (*y* axis) principal components. **(B)** The axes correspond to the third (*x* axis) and the fourth (*y* axis) principal components. *B. subtilis* (Bs), *M. marinum* (Mm), *K. pneumoniae* non-pathogenic strain (KpGe) and pathogenic mutant (Kp21), and *M. luteus* (Ml).

In order to visualize the similarities between the transcription profiles observed in each condition, as well as the reproducibility of the observed changes, we performed a Principal Component Analysis (PCA) using data from each independent experiment. To improve the relevance and consistency of the clustering, the data were first corrected for date-batch effect before plotting all seven subpopulations (Figure 4). The transcriptome of each sample was projected on two-dimensional planes with axes corresponding to the first two pairs of principal components (PCs). PC1 and PC2 accounted for 38% of the variation in the dataset, whereas PC3 and PC4 explained an additional 17% of the dataset variance (Figure 4). A significant degree of similarity between biological replicates was evident in the PCA plots, since the results of each condition in different experiments appeared clustered. The first two principal components revealed that the *B. subtilis* and *K. pneumoniae* (KpGe and Kp21) samples formed two separate clusters, distinct from the four other conditions (control, *M. luteus*, *M. marinum*, and folate) (Figure 4A), indicating that there are profound differences in the transcription profiles induced by *K. pneumoniae* and *B. subtilis* bacteria. Remarkably the two strains of *K. pneumoniae* analyzed induced similar transcriptional adaptations (Figure 4A). The principal components 3 and 4 clustered *M. marinum* samples together away from the other samples (Figure 4B). As would be expected from the analysis described above, exposure to *M. luteus* or to folate did not generate a significant global change in the transcriptional profile of *D. discoideum*. Indeed, to validate these results, we plotted the data on a tSNE plot and we observed that the clustering was similar to that reported above (Supplementary Figure S2).

Together these results suggest that *D. discoideum* mounts a robust transcriptional response when exposed to *M. marinum*, *K. pneumoniae*, and *B. subtilis*. The responses elicited by these three very different bacteria (mycobacteria, Gram-negative and Gram-positive bacteria, respectively) differ markedly from each other.

### Functional Categorization of DE Genes Shows Bacteria-Specific Enriched Pathways

**FIGURE 5:**
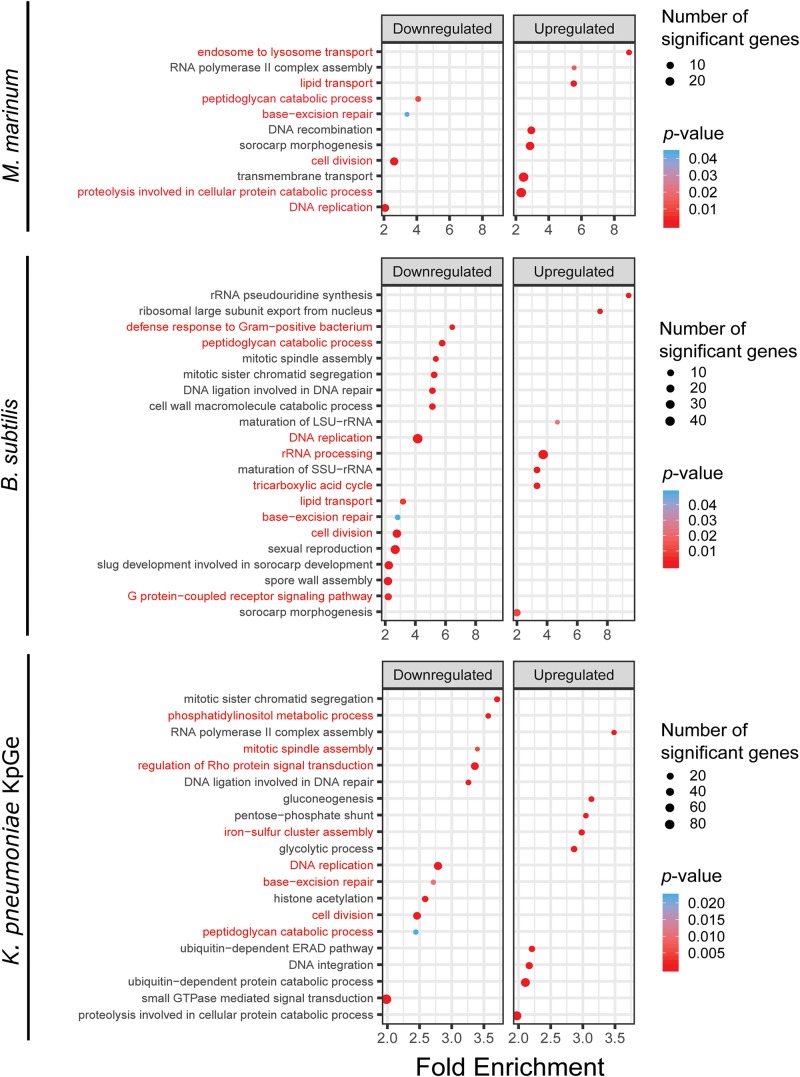
TopGO pathway analysis. GO term analysis was used to identify pathways related to biological processes overrepresented in genes that constitute the *D. discoideum* response to *M. marinum*, *B. subtilis* and *K. pneumoniae* exposure relative to mock controls. Genes that were differentially expressed by ≥1.5-fold with an adjusted *p*-value ≤ 0.05 were used as input for the topGO analysis, with up- and down-regulated genes considered separately. For each pathway, the fold enrichment (*x*-axis) was plotted against its *p*-value (color coding of the dots) and the count of significant genes in the respective GO term (dot size).

To reveal the biological pathways that underline the specific signatures visualized by PCA, we performed GO analysis based on a broader set of DE genes by using a less stringent cut off for the fold change (≥| 1.5|) and the adjusted *p*-value (≤0.05). The 5365 DE genes were subjected to a topGO enrichment analysis of biological processes. To more finely appreciate the pathways that are stimulated or repressed, DE genes were separated in up and down categories for the analysis (Supplementary Table S3 and Supplementary Figure S3). The GO enrichment data were selected based on their enrichment factor (EF) (≥2) and *p*-value (≤0.05) (Figure 5). Some biological processes appeared relatively consistently down-regulated in response to the different bacteria and are, for the most part, associated with cell growth (cell division, peptidoglycan catabolic process, base-excision repair and DNA replication) (Box 1 in Supplementary Figure S3). To the contrary, no functional group was up-regulated in common in response to all types of bacteria. Interestingly, and corroborating the PCA analysis, the transcriptome signatures varied markedly between the different types of bacteria. For example, in the presence of *M. marinum*, *D. discoideum* induces the transcription of genes related to endosome to lysosome transport (including the ESCRT machinery), lipid transport and proteolysis (including ubiquitin-mediated degradation) (Figure 5 and Box 2 in Supplementary Figure S3). This data suggests that *D. discoideum* senses *M. marinum* as a stress or that *M. marinum* induces a starvation-like condition (Hanna et al., 2019). In contrast to *M. marinum*, the lipid transport group was down-regulated in the presence of *B. subtilis* (Figure 5). The most enriched up-regulated genes in response to *B. subtilis* fell into RNA-related functional groups (rRNA processing, ribosomal large subunit export from nucleus), and the TCA cycle. In parallel, mitotic spindle assembly, defense to Gram-positive bacteria, and the GPCR signaling pathway were down-regulated (Figure 5). On the other hand, the transcriptomic response of *D. discoideum* to *K. pneumoniae* KpGe differed from that induced by other bacteria, and groups related to histone acetylation and signaling-related biological processes groups (small GTPase-mediated signal transduction, Rho, and phosphatidylinositol metabolic process), were down-regulated (Figure 5). The most enriched biological processes groups were related to cell metabolism such as the pentose-phosphate shunt, a parallel pathway to glycolysis and a precursor for the synthesis of nucleotides, detoxification (iron-sulfur cluster assembly), and ubiquitin dependent catabolic process (Figure 5).

### Does Transcriptional Profiling Identify Genes Implicated in Bacteria Sensing?

**FIGURE 6:**
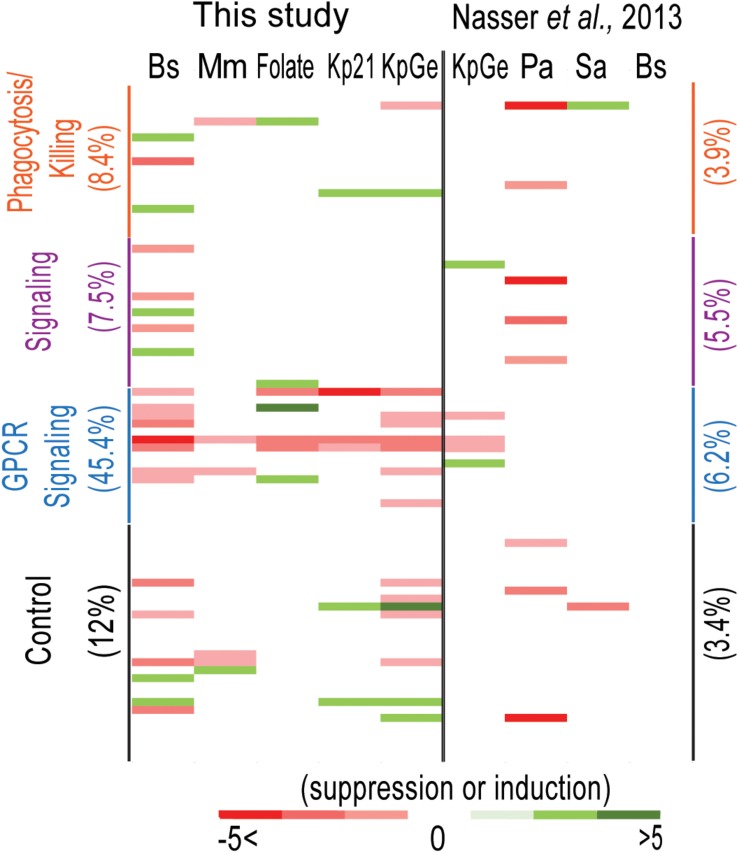
Analysis of the expression level of functionally significant genes. We compared the expression level of genes implicated in bacteria recognition (sensing and signaling), phagocytosis and killing (Supplementary Table S4) and 30 randomly selected genes with unknown function (control) under each of the studied conditions obtained in our study. A similar analysis was performed on the data published by Nasser and collaborators (Nasser et al., 2013). *B. subtilis* (Bs), *K. pneumoniae* LM21 (Kp21), *K. pneumoniae* (KpGe), *M. marinum* (Mm), *P. aeruginosa* (Pa), *S. aureus* (Sa), and folate. The percentage of genes that vary under each condition is indicated between brackets. Each variable for each bacterium was indicated with a color code varying from –5 to 5.

One expects a cell to upregulate the expression of useful genes to respond to changes in its environment. Accordingly, one may for example expect genes upregulated in the presence of *K. pneumoniae* to be useful during sensing, ingestion or killing of *K. pneumoniae*. To verify this hypothesis, we analyzed in the transcriptional datasets the expression of genes that have been previously implicated in bacterial recognition (sensing and signaling), phagocytosis and intracellular killing (Figure 6 and Supplementary Table S4). Note that, contrary to the unbiased analysis presented in Figure 5, this analysis focused on a biased list of genes deemed by the authors to be particularly relevant. As detailed in the corresponding references (Supplementary Table S4), this list includes genes for which gene inactivation has been shown to result in defective phagocytosis, intracellular killing, sensing or intracellular signaling, or genes that seem likely to be involved in these processes (e.g., lysozyme involved in killing of bacteria, or GPCRs involved in sensing of extracellular signals). When cells were exposed to bacteria or folate, genes implicated in phagocytosis/killing and signaling were differentially expressed in 8.4% and 7.5% of the situations analyzed, respectively, a figure even lower than that observed upon analysis of 30 control genes of unknown function (12% of differential expression). When the average fold change was analyzed, a similar result was obtained (0. 12-, 0. 11-, and 0.20-fold change for phagocytosis/killing genes, sensing genes and control genes, respectively) (Supplementary Table S4). Similar results were observed using 30 control genes selected randomly from the amoeba genome or using all the genes detected in this study (Supplementary Table S4). This observation suggests that to the best of our knowledge, genes that are differentially expressed in the presence of bacteria or folate do not have a higher probability of being involved in phagocytosis, intracellular killing or signaling than control genes (Supplementary Table S4).

We also analyzed the expression of glutamate receptor-like proteins, known as Grl proteins (Heidel et al., 2011). Grls are known to act as receptors for extracellular signals (Thomas et al., 2018), and *grl*G and *grl*L have been proposed to act as folate receptors and LPS (Pan et al., 2018; Thomas et al., 2018). We observed that the expression of 45.4% of these genes vary in particular *grlA*, *G, H*, and *L* (Figure 6 and Supplementary Table S4). The average fold change for this group of genes (1.04) was also much higher than in the controls. We speculate that our RNA-seq experiments allow to identify genes implicated potentially in bacterial sensing.

Recently, Nasser and collaborators performed RNA-seq experiments, in which they compared the expression levels of *D. discoideum* genes grown in the presence of four different bacteria: two Gram-positive bacteria *B. subtilis* and *Staphylococcus aureus*, and two Gram-negative bacteria *K. pneumoniae* KpGe and *P. aeruginosa* (Nasser et al., 2013). It should be stressed that these experiments were conducted and analyzed in a very different manner than in the present study: *D. discoideum* cells were plated on a lawn of the indicated bacteria on Agar plates and allowed to grow for 16 h, i.e., for several generations. Variations of gene expression levels were calculated as the log2 ratio of averaged normalized mRNA abundance on that bacterial species to the maximum of the averaged scaled mRNA abundance on the other bacterial species (Nasser et al., 2013). It is thus almost impossible to carry out a direct comparison of the results obtained by us and by Nasser et al. (2013). We did, however, determine the expression levels of the selected set of amoebal genes in the RNA-seq experiments performed by Nasser et al. (2013). We found that differential expression of selected genes was observed in 3.9–6.25% of selected genes implicated in phagocytosis/killing/GPCRs (average fold change 0.08–0.15) (Figure 6 and Supplementary Table S4). However, using different sets of control genes, we observe a differential expression ranging from 3.44% (for genes with unknown functions) to 16.4% (average for all genes detected). Given these results, it is difficult to conclude if genes implicated in bacteria recognition (sensing and signaling), phagocytosis or killing are more represented than irrelevant genes among differentially expressed genes identified in this study.

## Discussion

*Dictyostelium discoideum* amoebae reside in soil environments that are inhabited by thousands of bacterial species (Curtis et al., 2002). It is unclear how amoebae cope with such a diversity and how they elaborate specific physiological responses to feed upon different bacteria. A detailed understanding of the amoebal response should increase our understanding of the interactions between amoebae and bacteria and may reveal novel antibacterial strategies in eukaryotes. In this study, we analyzed by RNA-seq the transcriptome of *D. discoideum* cells exposed to various bacteria. Our results show that *D. discoideum* responds very differently when exposed to different bacteria, both quantitatively and qualitatively. Some bacteria elicit virtually no transcriptional response (*M. luteus*), others a moderate response (*K. pneumoniae*, *M. marinum*), and still others a strong response (*B. subtilis*). The amoebal response to each bacterium is largely specific. Although it has been proposed that secreted bacterial folate is an important signal sensed by *D. discoideum*, response to folate was much more limited than response to bacteria. These observations suggest that beyond folate many other bacterial molecules are recognized by *D. discoideum*.

We confirmed the RNA-seq data obtained in the current study, by testing the expression of 24 selected genes using qRT-PCR. Of these 24 genes, 22 were found to vary in the manner predicted by RNA-Seq analysis when cells were exposed to *K. pneumoniae* or *B. subtilis*. This confirmed that the RNA-seq data obtained in the current study is a reliable reflection of the transcriptional adaptation of *D. discoideum* to various bacteria. Defining this set of genes specifically regulated in the presence of *K. pneumoniae* and *B. subtilis* provides a tool that can be used in the future to test whether various *D. discoideum* mutants are capable of adapting their transcriptional profiles to the presence of these bacteria.

Phagocytosis is the major mechanism by which amoebae digest intracellular bacteria with the purpose of nutrient acquisition. However, pathogenic bacteria, including *M. marinum*, have evolved mechanisms to escape degradation in the phagosomes. In *D. discoideum*, as in other phagocytes, some bacteria escape from the phagosome by inducing membrane damage (Cardenal-Muñoz et al., 2017). In the present experiments, the exposure of *D. discoideum* to *M. marinum* triggered some specific transcriptional changes that were also revealed in a time-resolved RNA-Seq profiling of the major steps of infection (Hanna et al., 2019). Indeed, the data clearly identify signatures specific to an *M. marinum* infection, with an up-regulation of many host defense pathways, including the ESCRT-mediated repair of the membrane of the mycobacteria-containing vacuole (López-Jiménez et al., 2018) as well as both lysosomal and autophagy-related degradation pathways (Cardenal-Muñoz et al., 2017; Hanna et al., 2019). Another major facet of the *D. discoideum*/*M. marinum* interaction is nutrient supply. Inside their hosts, intracellular bacteria are restricted to a limited supply of nutrients, and to drive their proliferation they exploit suitable energy sources, which in the case of *M. marinum* is the host cells’ lipids (Barisch and Soldati, 2017). Although the pathways used by *M. marinum* to access the host lipids are poorly understood, our data suggests that this step could be facilitated by ABC lipid transporters which are upregulated in our transcriptome. Finally, 11 out of the 20 most down-regulated genes in cells exposed to *M. marinum* were hsp20-containing heat shock proteins with Hspg1 showing the highest fold change (=36). At this stage, we lack a functional interpretation for this observation, but a similar phenotype has been observed upon exposure of AGS gastric adenocarcinoma cells to *H. pylori* (Lang et al., 2016). This observation may thus warrant further scrutiny.

Can this study be used to select genes potentially implicated in the response to bacteria, for example genes involved in phagocytosis, intracellular killing or intracellular signaling? There are both practical and conceptual difficulties in analyzing the datasets generated in this and in other studies of *D. discoideum* transcriptional response to bacteria (Farbrother et al., 2006; Carilla-Latorre et al., 2008; Sillo et al., 2008; Nasser et al., 2013). First, different studies were conducted under different conditions, used different technologies to determine gene expression patterns, and different strategies to analyze the data collected. Second, in all these studies (Farbrother et al., 2006; Carilla-Latorre et al., 2008; Sillo et al., 2008; Nasser et al., 2013), including the present study, it is not possible to disentangle completely metabolic adaptation from bacterial sensing: bacteria are both a source of nutrients, and a source of extracellular signals. In the current study *M. luteus* did not induce any major change in gene expression and this observation suggests that the physiology and transcriptome of *D. discoideum* cells is not perturbed in this setup simply by the fact that bacteria represent an alternative food source to HL5c. Third, it is not easy to relate the observed changes to the situation(s) encountered by *D. discoideum* in their natural habitat. For example, when meeting a *K. pneumoniae* bacterium, does *D. discoideum* adapt its gene expression pattern to eat and kill efficiently *K. pneumoniae*, or all Gram-negative bacteria? Does *D. discoideum* on the contrary avoid phagocytosis and upregulate genes allowing escape, because some Gram-negative bacteria are pathogenic?

With all these limitations in mind, in the current study we found that genes known or strongly suspected to be involved in phagocytosis, intracellular killing or cell motility are not more differentially expressed than control genes upon encountering bacteria. On the contrary, Grls, in particular *grlA*, *G, H*, and *L* are highly regulated (mostly repressed) in *D. discoideum* exposed to bacteria or folate. Remarkably, this short list includes the two receptors (*grlG/far2* and *grlL/far1*) previously proposed to act as receptors for folate and bacterial LPS (Leiba et al., 2017; Pan et al., 2018; Thomas et al., 2018). It is common to observe down-regulation of a receptor upon engagement of its ligand as a means to down-regulate the cellular response. In this sense our observations are in agreement with the notion that *grl*G and *grl*L are receptors for bacterial products. It may be interesting to compare the role of these four gene products during the encounter of *D. discoideum* with other bacteria, for example by generating and comparing the corresponding gene knockout strains.

## Data Availability Statement

RNA-seq data that support the findings of this study have been deposited in Gene Expression Omnibus (GEO) with the accession code GSE144912.

## Author Contributions

PC and TS conceived and designed the study, with input from all other co-authors. AM performed the RNA-Seq experiments. OL performed the qRT-PCR experiments. OL, FB, MP, JN, JP, and PC performed the bioinformatic analysis. OL, NH, and PC wrote the manuscript. All authors read and contributed to the manuscript.

## Conflict of Interest

The authors declare that the research was conducted in the absence of any commercial or financial relationships that could be construed as a potential conflict of interest.

## Supplementary Material

FIGURE S1 **(A)** Bacterial growth in the presence or the absence of *D. discoideum. B. subtilis* (Bs) and *K. pneumoniae* (KpGe). **(B)** Internalization of fluorescent *B. subtilis* (Bs) or *K. pneumoniae* (KpGe) by *D. discoideum* after 4 h of coculture. Scale bars: 2 μm.

FIGURE S2 t-SNE visualization of the data reduced to 2 dimensions. Each dot represents one sample in an independent experiment. The expression profiles were adjusted for the experimental batches. *B. subtilis* (Bs), *M. marinum* (Mm), *K. pneumoniae* non-pathogenic strain (KpGe) and pathogenic mutant (Kp21), and *M. luteus* (Ml).

FIGURE S3 Heat map of the enriched biological processes in the topGo analysis. The samples descriptions are identical to Figure 5.

TABLE S1 List of all RNAs identified in this study.

TABLE S2 Number of differentially expressed genes observed in this study.

TABLE S3 List of biological processes groups identified by the topGo analysis.

TABLE S4 Expression levels of genes implicated in bacteria recognition (sensing and signaling), phagocytosis, and killing.
