# Supplementary figures and images for: Transcriptional Responses of Dictyostelium discoideum Exposed to Different Classes of Bacteria
Source: Front Microbiol. 2020 Mar 10;11:410. doi: 10.3389/fmicb.2020.00410 (PMC7078664; doi:10.3389/fmicb.2020.00410)

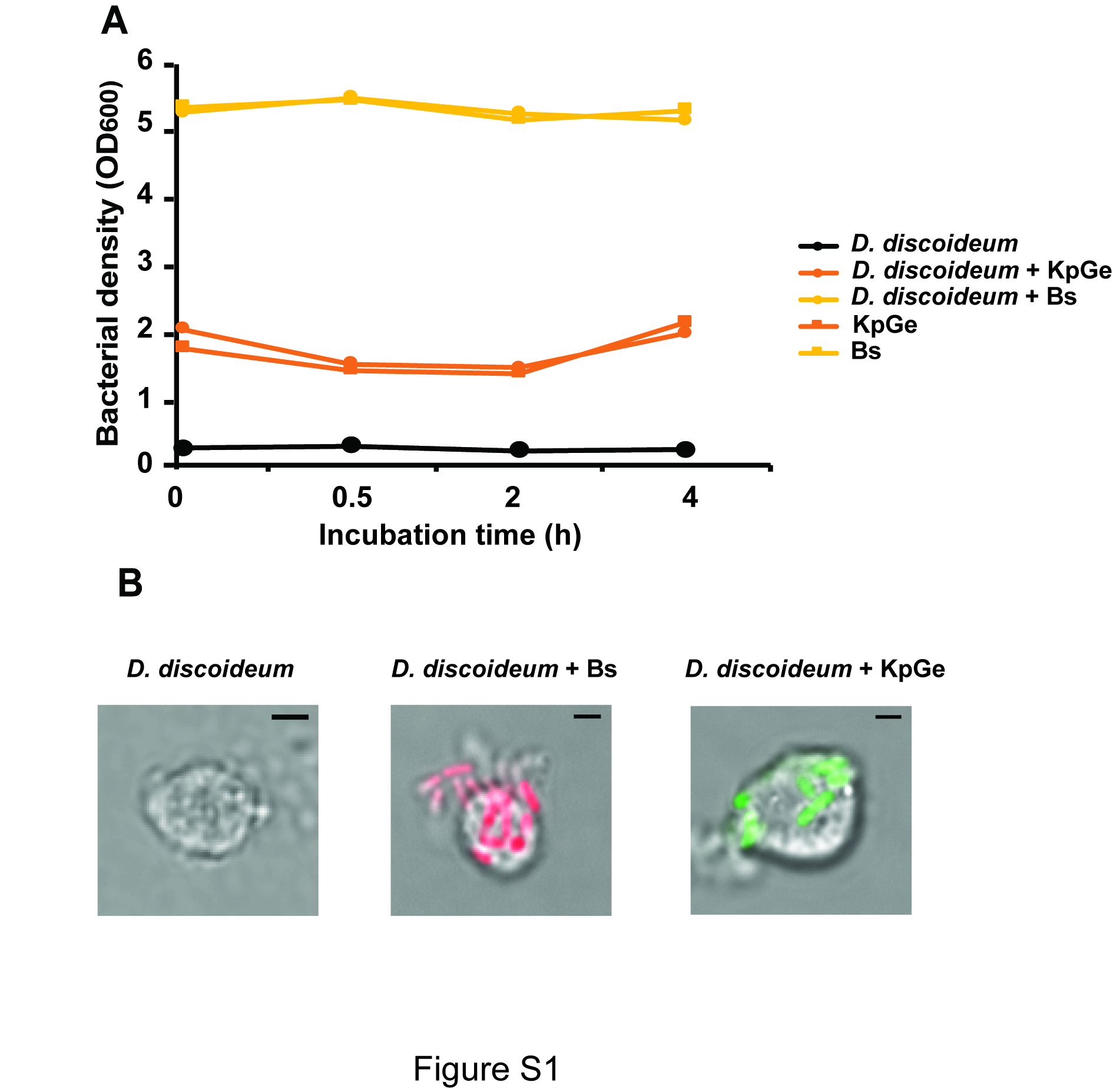

Supplement: FIGURE S1 — (A) Bacterial growth in the presence or the absence of D. discoideum. B. subtilis (Bs) and K. pneumoniae (KpGe). (B) Internalization of fluorescent B. subtilis (Bs) or K. pneumoniae (KpGe) by D. discoideum after 4 h of coculture. Scale bars: 2 μm. [file Image_1.TIF]

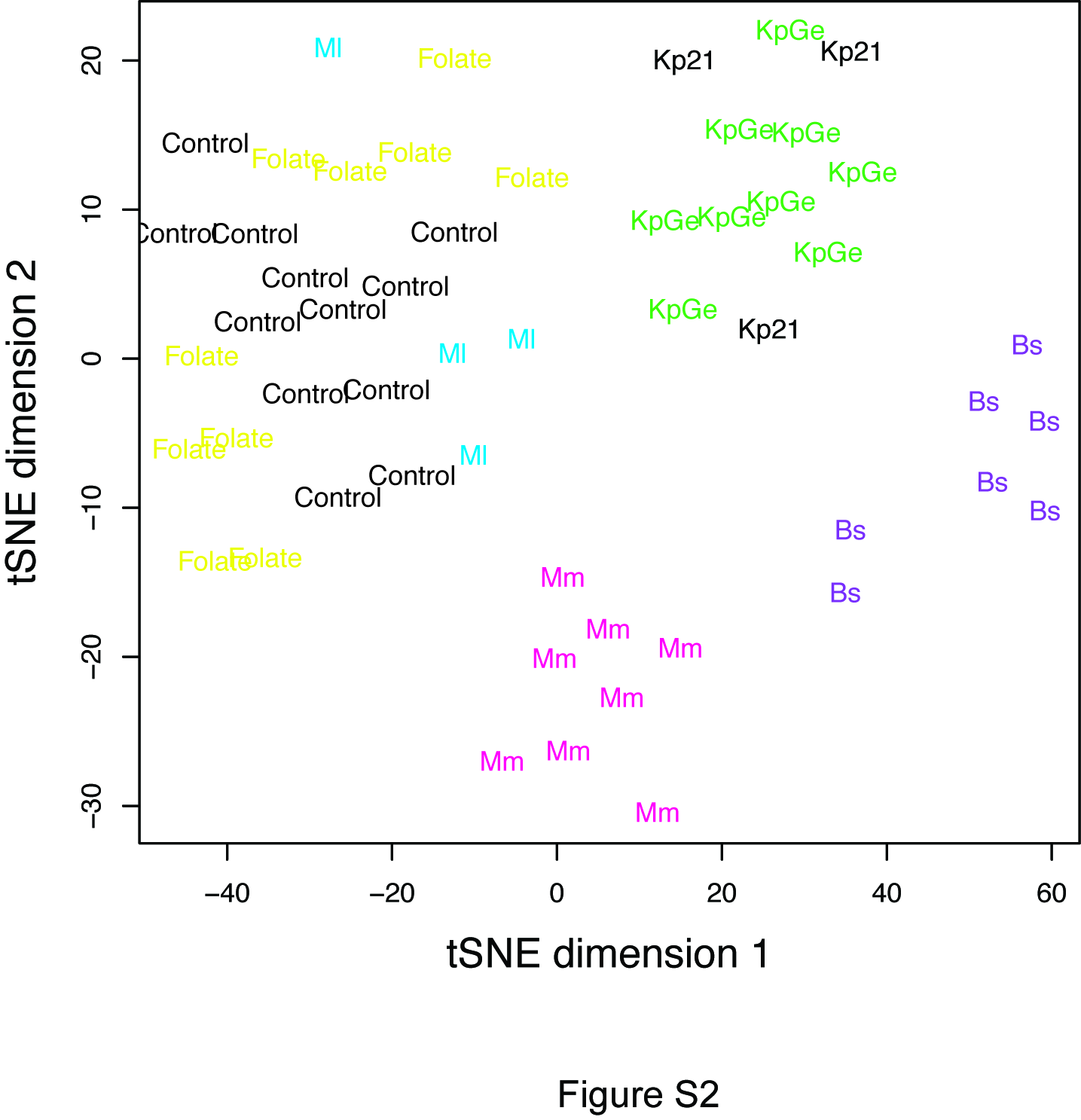

Supplement: FIGURE S2 — t-SNE visualization of the data reduced to 2 dimensions. Each dot represents one sample in an independent experiment. The expression profiles were adjusted for the experimental batches. B. subtilis (Bs), M. marinum (Mm), K. pneumoniae non-pathogenic strain (KpGe) and pathogenic mutant (Kp21), and M. luteus (Ml). [file Image_2.TIF]

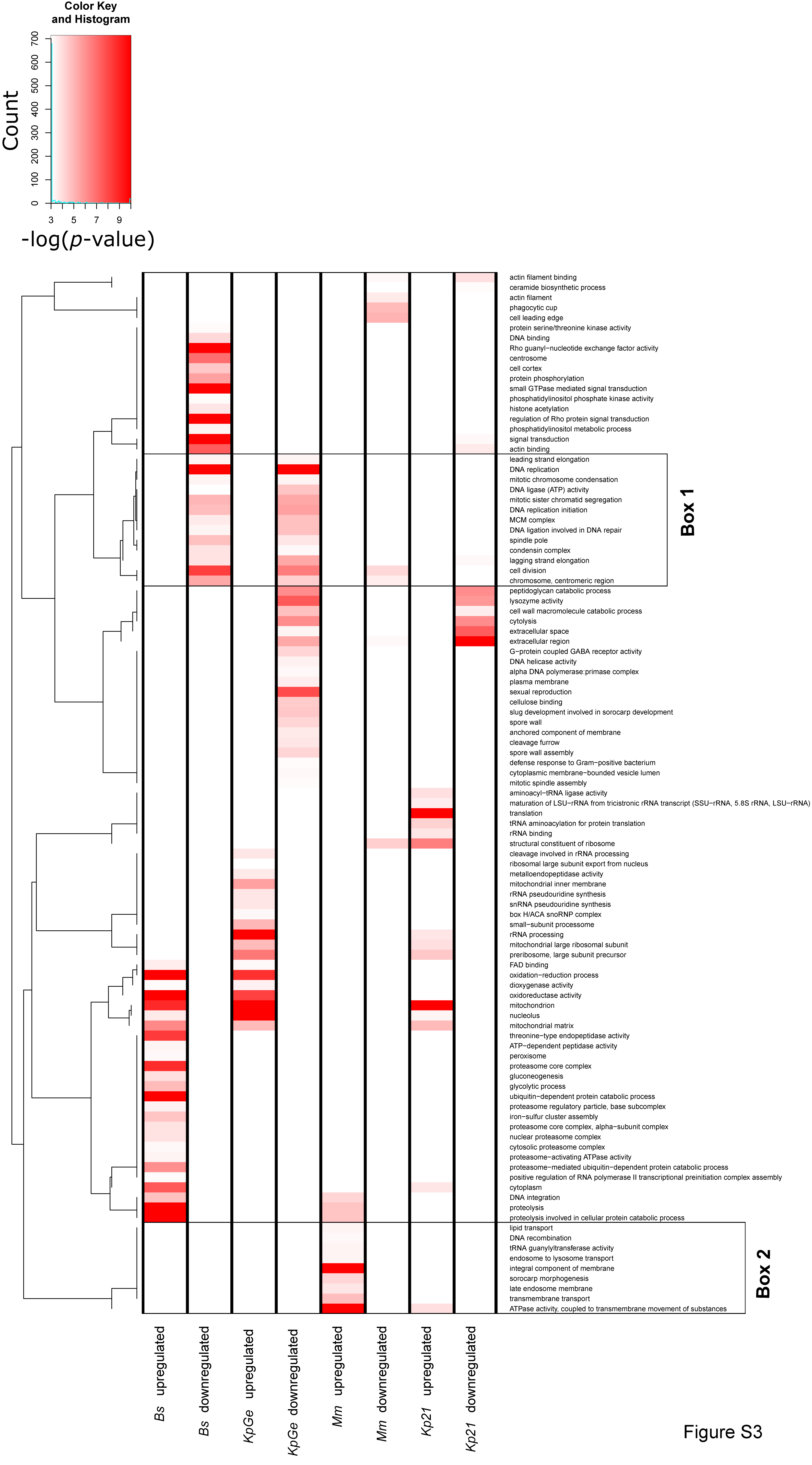

Supplement: FIGURE S3 — Heat map of the enriched biological processes in the topGo analysis. The samples descriptions are identical to Figure 5. [file Image_3.TIF]
